# *Streptococcus pneumoniae* Coinfection Is Correlated with the Severity of H1N1 Pandemic Influenza

**DOI:** 10.1371/journal.pone.0008540

**Published:** 2009-12-31

**Authors:** Gustavo Palacios, Mady Hornig, Daniel Cisterna, Nazir Savji, Ana Valeria Bussetti, Vishal Kapoor, Jeffrey Hui, Rafal Tokarz, Thomas Briese, Elsa Baumeister, W. Ian Lipkin

**Affiliations:** 1 Center for Infection and Immunity, Mailman School of Public Health, Columbia University, New York, New York, United States of America; 2 Instituto Nacional de Enfermedades Infecciosas, Administracion Nacional de Laboratorios e Institutos de Salud “Dr. Carlos G. Malbrán”, Buenos Aires, Argentina; University of British Columbia, Canada

## Abstract

**Background:**

Initial reports in May 2009 of the novel influenza strain H1N1pdm estimated a case fatality rate (CFR) of 0.6%, similar to that of seasonal influenza. In July 2009, however, Argentina reported 3056 cases with 137 deaths, representing a CFR of 4.5%. Potential explanations for increased CFR included virus reassortment or genetic drift, or infection of a more vulnerable population. Virus genomic sequencing of 26 Argentinian samples representing both severe and mild disease indicated no evidence of reassortment, mutations associated with resistance to antiviral drugs, or genetic drift that might contribute to virulence. Furthermore, no evidence was found for increased frequency of risk factors for H1N1pdm disease.

**Methods/Principal Findings:**

We examined nasopharyngeal swab samples (NPS) from 199 cases of H1N1pdm infection from Argentina with MassTag PCR, testing for 33 additional microbial agents. The study population consisted of 199 H1N1pdm-infected subjects sampled between 23 June and 4 July 2009. Thirty-nine had severe disease defined as death (n = 20) or hospitalization (n = 19); 160 had mild disease. At least one additional agent of potential pathogenic importance was identified in 152 samples (76%), including *Streptococcus pneumoniae* (n = 62); *Haemophilus influenzae* (n = 104); human respiratory syncytial virus A (n = 11) and B (n = 1); human rhinovirus A (n = 1) and B (n = 4); human coronaviruses 229E (n = 1) and OC43 (n = 2); *Klebsiella pneumoniae* (n = 2); *Acinetobacter baumannii* (n = 2); *Serratia marcescens* (n = 1); and *Staphylococcus aureus* (n = 35) and methicillin-resistant *S. aureus* (MRSA, n = 6). The presence of *S. pneumoniae* was strongly correlated with severe disease. *S. pneumoniae* was present in 56.4% of severe cases versus 25% of mild cases; more than one-third of H1N1pdm NPS with *S. pneumoniae* were from subjects with severe disease (22 of 62 *S. pneumoniae*-positive NPS, p = 0.0004). In subjects 6 to 55 years of age, the adjusted odds ratio (OR) of severe disease in the presence of *S. pneumoniae* was 125.5 (95% confidence interval [CI], 16.95, 928.72; p<0.0001).

**Conclusions/Significance:**

The association of *S. pneumoniae* with morbidity and mortality is established in the current and previous influenza pandemics. However, this study is the first to demonstrate the prognostic significance of non-invasive antemortem diagnosis of *S. pneumoniae* infection and may provide insights into clinical management.

## Introduction

On June 11, 2009, the World Health Organization (WHO) declared a pandemic outbreak of respiratory illness associated with the novel influenza A (H1N1) virus (H1N1pdm). Infection with H1N1pdm was considered mild; however, the strain seemed to be highly transmissible. Based on a study in the community of La Gloria, Mexico, where the virus was first detected early in 2009, and worldwide surveillance data and mathematical modeling, the CFR was estimated to be 0.6% [1]. The first case in Argentina was reported on May 17, 2009; by July 16, 2009, just two months later, the number of cases in Argentina totaled 3056, with 137 deaths, representing a computed CFR of 4.5% [2].

Although we could not exclude the possibility that this elevated CFR reflected underreporting of milder infections, the alternative, a *bona fide* increase due to differences at the level of host or pathogen, might have global implications. Risk factors commonly associated with development of severe disease, including advanced age, chronic illnesses (diabetes, asthma, obesity) or immunosuppression (other immune-mediated diseases, immunomodulatory therapies, pregnancy), were not present at increased frequency in Argentina. Another potential explanation for the higher CFR was a change in the virus, including reassortment with a more virulent strain, development of resistance to antiviral therapies, or genetic drift, resulting in a more virulent phenotype. Complete genome sequencing of 26 samples representing both severe and mild cases of disease from Argentina did not reveal evidence consistent with development of a more virulent phenotype (data not shown). A third factor we considered was bacterial and viral coinfection. Although disease severity in previous pandemics has been attributed to bacterial superinfection [3], early reports among H1N1pdm-infected individuals in developed countries failed to correlate severe disease with coinfection [2], [4], [5].

To evaluate the contribution of coinfection to the mortality observed in Argentina, we examined 199 cases of previously-diagnosed H1N1pdm infections from Argentina, including 39 cases classified as severe and 160 cases categorized as mild, using MassTag PCR, a multiplex PCR system that assesses 30 to 40 microbial agents in a single reaction [6].

## Results

NPS specimens from 199 H1N1pdm-positive patients, collected between June 23 and July 4 during the course of an outbreak investigation of pandemic H1N1 influenza in Argentina, were analyzed by MassTag PCR for the presence of potential sources of coinfection, including 33 viral and bacterial respiratory pathogens. Prior to initiating MassTag PCR studies, the presence of H1N1pdm was confirmed for all samples with the WHO-approved Real Time PCR H1N1pdm assay [7].

Subjects included 39 patients classified with severe disease, based on hospitalization (n = 19) or death (n = 20), and 160 patients with mild disease, who presented to ambulatory clinics. Five patients had medical conditions associated with increased risk of severe disease following influenza infection (asthma, malnutrition, pregnancy, obesity, immunosuppression; n = 1 each). Four of these patients (80%) had severe disease; 1 had mild disease (*p* = 0.0056).

To examine whether the age groups typically associated with greater risk of influenza-related complications – i.e., young children and the elderly [8] – were also at higher risk for more severe disease in the context of H1N1pdm, we compared the severity of disease in subjects <6 years of age or >55 years of age (high risk age group) with that found in subjects 6 to 55 years of age (low risk age group; age data available for 181 subjects). Although the mean age of severe and mild disease subjects was similar, severe disease occurred in 14 of 26 (53.8%) of high risk age group subjects as compared with 20 of 155 (12.9%) of the low risk age group subjects (Fisher's exact test, *p*<0.0001; **Table 1**). However, of the 34 patients with severe disease for whom age data were available, 20 (58.8%) were from the low risk age group (Fisher's exact test, *p*<0.0001). There was a trend toward a higher prevalence of severe disease in males (20 of 80, or 25.0%) as compared with females (15 of 104, or 14.4%; *p* = 0.09).

**Table 1:** Characteristics of H1N1pdm influenza subjects.

SUBJECT CHARACTERISTIC		DISEASE SEVERITY		ALL SUBJECTS
		Severe	Mild	
		(total n = 39)	(total n = 160)	(total n = 199)
		n (%)	n (%)	n (%)
AGE	Subjects with available age data	34 (87.2)	147 (91.8)	181 (91.0)
	in years, mean±SD	27.8±21.6*	23.9±15.5	24.7±16.8
AGE RISK CATEGORY	High risk: <6 or >55 years	14 (41.2)**	12 (8.2)	26 (14.4)
	Low risk: 6–55 years	20 (58.8)	135 (91.8)	155 (85.6)
SEX	Subjects with available sex data	35 (89.7)	149 (93.1)	184 (92.5)
	Female	15 (42.9)§	89 (59.7)	104 (56.5)
	Male	20 (57.1)	60 (40.3)	80 (43.5)
ANTI-MICROBIAL STATUS	Subjects with available drug data	29 (74.3)	91 (56.9)	120 (60.3)
	No antimicrobial drugs	3 (10.3)¶	2 (2.2)	5 (4.2)
	Antiviral drugs only	10 (34.5)	86 (94.5)	96 (80.0)
	Antibacterial drugs only	13 (44.8)	1 (1.1)	14 (11.7)
	Antivirals + antibacterials	3 (10.3)	2 (2.2)	5 (4.2)
MEDICAL RISK FACTOR		4 (10.3)#	1 (0.6)	5 (2.5)

* Mann-Whitney U, *p* = ns.

** *p*<0.0001.

§ *p* = 0.09.

¶ *p*<0.0001.

# *p* = 0.0056.

Data concerning antiviral and antibiotic therapy were available for 120 subjects. Risk of severe disease was diminished in subjects who received only oseltamivir. Of 96 subjects receiving oseltamavir alone, 10 (10.4%) had severe disease. In contrast, 13 of 14 patients (92.9%) who received antibiotics without antiviral medication had severe disease (*p*<0.0001).

### MassTag PCR assays

MassTag PCR detected H1N1pdm and at least one additional potential respiratory pathogen in 152 of 199 samples (76.4%). Co-infecting agents included *S. pneumoniae* (n = 62); *H. influenzae* (n = 104); human respiratory syncytial virus (RSV) A (n = 11) and B (n = 1); human rhinovirus (HRV) A (n = 1) and B (n = 4); human coronavirus (HCoV) −229 (n = 1) and -OC43 (n = 2); *K. pneumoniae* (n = 2); *A. baumannii* (n = 2); *S. marcescens* (n = 1); and *S. aureus* (n = 35) and MRSA (n = 6) (**Table 2**). In all cases, the only influenza virus found was H1N1pdm. The presence of *S. pneumoniae* was associated with severe disease. *S. pneumoniae was* detected in 56.4% of severe cases, but only 25.0% of mild cases; more than one-third of H1N1pdm NPS with *S. pneumoniae* were from subjects with severe disease (22 of 62 *S. pneumoniae*-positive NPS, *p* = 0.0004; **Table 2**). *H. influenzae* infection was frequent in the study population – 104 of all 199 cases (52.3%) harbored the agent – but was not increased among subjects with severe disease. Whereas 95 of the 160 mild H1N1pdm cases (59.4%) were positive for this bacterium, *H. influenzae* was only detected in 9 of 39 severe H1N1 cases (23.1%, *p*<0.0001). Other bacterial agents were detected in 45 cases, including *S. aureus* (n = 35), MRSA (n = 6), *K. pneumoniae* (n = 2), *S. marcescens* (n = 1), and *A. baumannii* (n = 2). Of the 45 subjects with one or more bacterial respiratory pathogens other than *S. pneumoniae* or *H. influenzae* in their NPS samples, 44 had mild disease (97.8%; *p* = 0.0004).

**Table 2:** Coinfection patterns in severe and mild H1N1pdm influenza.

AGENT(S) DETECTED	DISEASE SEVERITY		ALL SUBJECTS
			(n = 199)
			n (%)
	Severe	Mild	
	(n = 39)	(n = 160)	
	n (%)	n (%)	
*S. pneumoniae*	22 (56.4)*	40 (25.0)	62 (35.5)
*H. influenzae*	9 (23.1)**	95 (59.4)	104 (52.3)
*S. aureus* (any)	1 (2.6)***	40 (25.0)	41 (20.6)
Methicillin-resistant *S. aureus* (MRSA)	0 (0.0)	6 (3.7)	6 (3.0)
Other bacterial respiratory pathogens‡	0 (0.0)	5 (3.1)	5 (2.5)
(*K. pneumoniae*, *S. marcescens*, *A. baumannii*)			
RSV A	6 (15.4)^¶^	5 (3.1)	11 (5.5)
Other viruses‡‡	1 (2.6)	8 (5.0)	9 (4.5)
(HRV, HCoV-229E, HCoV-OC43, RSV B)			
**Any coinfection**	15 (38.5)	110 (68.7)	125 (62.8)
Other bacterial agent ± *S. pneumoniae OR* Any virus in addition to H1N1			
Coinfection in addition to *S. pneumoniae* (n = 62)	*10 (25.6)*	*30 (18.7)*	*40 (20.1)*
Other coinfection without *S. pneumoniae* (n = 137)	*5 (12.8)^¶¶^*	*80 (50.0)*	*85 (42.7)*

* *p* = 0.0004; ** *p*<0.0001; *** *p* = 0.0008; ^¶^ *p* = 0.0085; ^¶¶^ *p* = 0.0017.

‡ No evidence of other bacterial respiratory pathogens in any subjects, including: *C. pneumoniae*; *L. pneumophila*; *M. pneumoniae*; *M. tuberculosis*; *N. meningitides*; *C. albicans*; *Enterobacter spp*; *Enterococcus spp*.; *Pseudomonas spp*.; *S. pyogenes*.

‡‡ No evidence of other viral respiratory pathogens in any subjects, including: strains of FLUAV other than H1N1pdm, FLUBV, HPIV 1-4, HMPV, HEV, HAdV. The *A. baumannii*-positive case was also positive for *S. aureus*.

The presence of RSV A, though relatively infrequent, was more common in severe (6 of 39 cases, 15.4%) than in mild disease (5 of 160 cases, 3.1%; *p* = 0.0085). Other viruses, including RSV B, HRV and HCoV, were also infrequent (9 of 199 cases, 4.5%). Only 1 such case (HRV B) had severe disease.

The significance of the relationship between *S. pneumoniae* and severe disease was restricted to patients in the low risk age group (*p*<0.0001; **Table 3**). To more closely examine the influence of the presence of *S. pneumoniae* in NPS samples on H1N1pdm severity as a function of age-associated risk, we pursued multivariable logistic regression analysis stratified by age risk group. A model adjusting for the presence in NPS of RSV A, the total number of agents detected in NPS, and the presence of a medical risk factor implicated in severe H1N1 outcomes was derived after ensuring that relationships among included variables were low (nonsignificant contingency coefficients ranging from 0.001 to 0.255). In the low age risk category (subjects aged 6 to 55 years), the adjusted OR for the contribution of *S. pneumoniae*-positivity to severe disease outcomes in this logistic regression model was 125 (95% CI, 16.95, 928.72; *p*<0.0001, **Table 4**. The presence of a risk-associated medical condition was associated with increased risk of severe disease (OR [95% CI], 15.31 [1.21, 193.49], *p* = 0.0358); the total number of agents detected was inversely related to severe disease outcomes (OR [95% CI], 0.11 [0.03, 0.38; *p* = 0.0005). Results of the logistic regression analysis were not significant for subjects with high age-associated risk (<6 or >55 years of age).

**Table 3:** Relationship of *S. pneumoniae* coinfection to H1N1 influenza disease severity, stratified by age risk category.

AGE RISK CATEGORY	PRESENCE OF AGENT	DISEASE SEVERITY		ALL SUBJECTS
		Severe	Mild	
		(n = 34)*	(n = 147)*	(n = 181)
**HIGH RISK**	*S. pneumoniae* (+)	7 (50.0)	7 (58.3)	14 (53.8)
Age <6 or >55 years (n = 26)	*S. pneumoniae* (−)	7 (50.0)	5 (41.7)	12 (46.1)
**LOW RISK**	*S. pneumoniae* (+)	13 (65.0)§	27 (20.0)	40 (25.8)
Age 6–55 years (n = 155)	*S. pneumoniae* (−)	7 (35.0)	108 (80.0)	115 (74.2)

* Restricted to subjects with age data.

§ *p*<0.0001.

**Table 4:** Relationship of presence of *S. pneumoniae* in NPS to H1N1pdm disease severity in 6-to-55 year-old subjects.

Variable	Unadjusted odds ratio (95% CI)	*p* value	Adjusted odds ratio (95% CI)*	*p* value
*S. pneumoniae* (+)	7.43 (2.70, 20.42)	0.0001	125.46 (16.95, 928.72)	<0.0001

* Adjusted for presence of RSV A in NPS, total number of agents detected in NPS, and presence of medical risk factor. Likelihood ratio of model fit, p<0.0001.

## Discussion

Secondary bacterial infections have been implicated in morbidity and mortality in H1N1 influenza [3]. Analysis of lung tissue sections from fatal 1918 influenza case materials frequently revealed histopathologic findings consistent with acute bacterial pneumonia [3]. Agents recovered by postmortem cultures of lung samples from 96 fatal 1918 influenza pandemic cases included *S. pneumoniae* (23.2%), *S. haemolyticus* (18.0%), *S. aureus* (7.7%) and *H. influenzae* (4.7%) [3].

In our study of Argentinean victims of H1N1pdm, the presence of *S. pneumoniae* in NPS predicts severe disease outcome. The risk associated with *S. pneumoniae* is particularly prominent in 6-to-55 year-old individuals. Indeed, severity of disease in this low risk group can be predicted with 90.97% accuracy via a multivariable logistic regression model that considers the presence of *S. pneumoniae* together with viruses other than influenza and a risk-associated medical condition.

In the first wave of pediatric H1N1pdm admissions in Birmingham, UK, only low rates of bacterial infection were found (10 out of 63 children, 15.8%) [4]. A case series of the first 18 H1N1pdm-infected patients hospitalized in Mexico City reported no evidence of coinfection with other common respiratory viruses [5]. In contrast to these initial reports based on clinical data, wherein coinfection was infrequent, recent postmortem analyses indicated lower respiratory tract infection in 22 out of 77 lethal 2009 H1N1pdm cases in the United States (29%) [9]; *S. pneumoniae* was implicated in 10 of these cases.

Synergistic pathogenesis is described between influenza virus and *S. pneumoniae*. Madhi and colleagues demonstrated that vaccination against *S. pneumoniae* reduces the frequency of pneumonia associated with influenza A, RSV and parainfluenza viruses [10]. In animal models, influenza neuraminidase has been shown to strip sialic acid residues to expose pneumococcal receptors on respiratory epithelium [11]. Indeed, the potency of neuraminidase is correlated with the capacity of an influenza virus strain to promote pneumonia [12].

Three practical implications emerge from our study. First, *S. pneumoniae* is important in the pathogenesis and prognosis of H1N1pdm-associated disease. Whether this effect is associated with *S. pneumoniae* sui generis or only with specific serotypes remains to be determined. Second, easily accessible samples such as NPS may be used as an index to risk of severe disease. Third, multiplex diagnostic methods like MassTag PCR can enable rapid detection of a broad spectrum of viral and bacterial agents and inform clinical care.

## Materials and Methods

### Data Collection

As is the routine during infectious disease outbreaks, specimens were collected at individual hospitals and point-of-care institutions in Argentina and submitted to the Administracion Nacional de Laboratorios e Institutos de Salud (ANLIS). Clinical information and samples submitted to ANLIS were assigned a new unique identifier to deidentify both the clinical data and biological samples; absence of any personally-identifying information was assured. Samples were submitted under deidentified codes to Instituto Nacional de Enfermedades Infecciosas (INEI) for H1N1pdm testing. Samples for this study were randomly selected at ANLIS by review of deidentified clinical information provided to ANLIS from hospital and point-of-care institutions on the basis of their review of charts in their own institutions. INEI and Columbia University (CU) were provided the following deidentified data: patient age, sex, presence of prior medical conditions known to be associated with greater morbidity and mortality after influenza infection (e.g., diabetes, chronic pulmonary disease, cardiovascular disease, obesity, immunosuppression, pregnancy), use of antimicrobial drugs, date of sample acquisition and geographic region of patient residence. Information about other potential factors associated with risk of severe H1N1 disease, including presence of passive tobacco smoke exposure, older siblings, or personal and/or familial atopic background, was unavailable. Both INEI and CU maintain approved Institutional Review Board (IRB) protocols for receipt and analysis of deidentified samples and their associated deidentified clinical data.

### Subjects and Specimens

Samples for this study were 199 H1N1pdm-positive NPS specimens collected at ANLIS in Buenos Aires, Argentina. Samples were randomly selected for analysis from two types of repository specimens maintained by ANLIS and collected during the course of standard H1N1pdm surveillance procedures from hospital and clinics in Argentina during the period 06/23/2009 to 07/04/2009: (1) NPS samples from mild H1N1pdm cases (n = 160), and (2) NPS samples from severe H1N1pdm cases (n = 39). Mild H1N1 cases were defined as ambulatory cases of confirmed H1N1 infection; severe H1N1 disease was defined on the basis of either death (n = 20) or severe pneumonia requiring hospitalization or mechanical assistance (n = 19). Prior to selection of samples for analysis, deidentified clinical information from hospital and clinics was used by approved ANLIS personnel to classify cases as mild or severe. Specimens were submitted to the INEI and archived at −70°C following testing for H1N1pdm by the WHO-approved Real Time PCR test [7]. During the same period (epidemiological weeks 25 and 26), 1496 RSV infections (15.6% out of 9595 virus-positive cases) were reported by the Argentinian national respiratory disease surveillance system in addition to 7867 FLUAV (82.0%; 6331 H1N1pdm, 1535 without typing information), 23 FLUBV (0.2%), 161 HPIV (1.7%) and 48 HAdV (0.5%) cases of severe acute respiratory disease [13].

To determine whether patterns of coinfection with other respiratory pathogens among H1N1pdm-positive patients with severe disease differed from coinfection patterns observed in those with mild disease, 39 NPS specimens were selected randomly from a total of 1769 severe cases, and studied along with 160 NPS selected randomly from among 1282 H1N1pdm-infected ambulatory (mild) cases. Both groups of samples were collected from 6/23/2009 to 7/4/2009.

### Laboratory Assays

Total RNA from NPS was obtained by acid guanidinium thiocyanate-phenol-chloroform extraction (TRI-Reagent, Molecular Research Center).

Samples were prepared and analyzed by MassTag PCR [6], [14] for 33 microbes using 3 panels targeting: generic influenza A virus (FLUAV), influenza B virus (FLUBV), RSV A, RSV B, HCoV OC43 and 229E, human parainfluenza (HPIV) 1-4, human metapneumovirus (HMPV), human enteroviruses (HEV), HRV (A, B and C), human adenovirus (HAdV), *C. pneumoniae*, *H. influenzae*, *L. pneumophila*, *M. pneumoniae*, *M. tuberculosis*, *N. meningitides*, *S. pneumoniae*, *A. baumannii*, *C. albicans*, *Enterobacter spp.*, *Enterococcus spp.*, *K. pneumoniae*, *S. aureus*, methicillin-resistant *S. aureus*, *Pseudomonas spp.*, *S. marcescens*, *and S. pyogenes* [6].

### Statistics

Group comparisons were conducted using nonparametric tests (Mann-Whitney U test) for continuous data deviating from normal distributions and Fisher's Exact Test for nominal data. Two-tailed tests for significance were pursued in all analyses. To examine H1N1pdm disease severity as a function of *S. pneumoniae* coinfection, we created a multivariable logistic regression model, stratified by age risk category (high risk, age <6 or >55 years; low risk, age 6–55 years). The outcome of interest was disease severity (severe disease: hospitalized or fatal H1N1pdm cases; mild disease: non-hospitalized, nonfatal community H1N1pdm cases). The model was adjusted for RSV A coinfection, the total number of bacterial or viral agents detected in NPS, and the presence of a medical risk factor. All variables were checked for correlation with each other and with the disease severity dependent variable by deriving contingency coefficients. The goodness-of-fit test was used to determine the fit of the logistic regression model to the data. StatView for Windows, version 5.0.1 (SAS Institute) and SPSS for Windows, version 17.0 (SPSS, Inc.) statistical software were employed for these analyses. Test levels for significance were *p*<0.05.
